# Gegenwissen. Die Neuen Sozialen Bewegungen in der Bundesrepublik und die Grundlagen ihrer Wirkung

**DOI:** 10.1007/s00048-022-00349-4

**Published:** 2022-10-12

**Authors:** Alexander von Schwerin

**Affiliations:** Max-Planck-Institut für Wissenschaftsgeschichte, Berlin, Deutschland

„Gegenwissen“ ist nicht nur ein nicht ganz leicht zu umgrenzendes historisches Thema, sondern provoziert heutzutage fast zwangsläufig Assoziationen zu Querdenkertum, Fake News-Aktivitäten, Anti-Intellektualismus und Wissenschaftsdefätismus. Die Grundlagen für solche Vergleiche sind jedoch dünn, wissen wir über das Gegenwissen in seiner historischen und gesellschaftlichen Vielfalt bislang doch nur wenig. Fest steht immerhin, dass alternatives und kritisches Wissen in der Vergangenheit die Wissenschaften, aber auch Medizin und Technik immer wieder herausforderten. Schaut man nur auf Deutschland im 20. Jahrhundert, so kommt schnell einiges zusammen: die weitläufigen Aktivitäten der Lebensreformbewegung rund um Ernährung, Gesundheit, Leib und Körper, die Medizinkritik der 1920er Jahre, Verbraucherschutz der 1950er, die Anti-Atom-Bewegung der späten 1950er und ab den 1970er Jahren, Sozialmedizin und Antipsychiatrie, Geschichtswerkstätten, Windmühlenbau in Landkommunen und private Projekte technisch gewiefter Solarentwickler. Und führt man sich allein die Kritik an den Gefahren der Atomenergie vor Augen, wird schon deutlich, dass alternatives und kritisches Wissen durchaus mit politischen und gesellschaftskritischen Positionen und Aktivitäten verbunden war. Die Wissenschaft selbst stand dabei nicht außen vor. Politischer Aktivismus von Wissenschaftler*innen ist ein historisch in letzter Zeit zunehmend thematisiertes Phänomen, wie die benachbarte *Special Section* zu diesem Heft aufzeigt (Germann et al. 2022). Die Grenzen zwischen Wissenschaft und anderen gesellschaftlichen Bereichen waren auch mit Blick auf alternatives und kritisches Wissen sehr durchlässig, wie diese *Special Section* zeigt.

Die Beiträge zu dieser *Special Section* konzentrieren sich auf kritisches und alternatives Wissen – als „Gegenwissen“ zusammengefasst – der Neuen Sozialen Bewegungen in der Bundesrepublik der 1960er bis 1990er Jahre und damit auf ein Phänomen, das so vielfältig und divers war wie jene Bewegungen (Rucht 2011: 41–43) selbst.

Plötzlich war das „Gegenwissen“ überall. Um das Jahr 1980 gründeten kritische Wissenschaftler*innen Zeitschriften und Wissenschaftsläden oder versuchten, ihr Wissen anderweitig den sozialen Bewegungen zur Verfügung zu stellen; Aktivist*innen veröffentlichen Handbücher und Informationsbroschüren; allerorts entstanden Initiativen, die das Alltagswissen und das „Wissen von unten“ für sich entdeckten und reklamierten (Stadler et al. 2020a).

Mit diesen Worten umreißen die Herausgeber*innen des kommentierten Quellenbandes „Gegen|Wissen“ das Phänomen, das untrennbar zur Geschichte der Neuen Sozialen Bewegungen zu gehören scheint, wenn nicht sogar deren konstitutive Grundlage und Formierungsbedingung bildete, dem die Geschichtsschreibung aber bislang wenig Beachtung geschenkt hat. Diesen Umstand nahmen die Autor*innen der vorliegenden *Special Section* im Jahr 2018 zum Anlass, auf der Jahrestagung der Gesellschaft für die Geschichte der Wissenschaften, der Medizin und der Technik (GWMT) einige Schlaglichter auf die Geschichte des Gegenwissens zu werfen.^1^ Eine Literaturübersicht kann diese kurze Einführung zur *Special Section* nicht leisten.

## Auf dem Weg in eine utopische Wissensgesellschaft

Seit den 1960er Jahren hat das Gegenwissen in den westlichen Industriestaaten einen starken Aufschwung genommen, mit einschneidender Bedeutung für das Verhältnis von Wissenschaft und Öffentlichkeit bis heute. Schon die Studierendenbewegung war eine Wissensbewegung, bedenkt man ihre obsessive Beschäftigung mit Theorie. Mehr noch, so scheint es, bauten die sozialen und alternativen Bewegungen der 1970er und 1980er Jahre auf einem alternativen – zudem stärker praxisorientierten – Wissenskorpus auf, der erst die Grundlage für ihre vielfältigen Aktivitäten schuf (zur Praxisorientierung: Hollstein 1979; Reichardt 2014; Falasca et al. 2018).

Der bewusst vollzogene Bruch mit dem Bestehenden war, wie die Bewegungsgeschichte herausgearbeitet hat, das vorherrschende Signum der Neuen Sozialen Bewegungen der 1970er und 1980er Jahre (Rucht 1994, 2011; Reichardt 2014). Die Entfremdung vom etablierten Wissenschaftsbetrieb vertiefte diesen Bruch noch. Studierenden‑, Alternativbewegung und immer breitere Teile der bürgerlichen Mittelschicht teilten ein grundsätzliches Misstrauen gegenüber den wissenschaftlichen Institutionen, das sich nicht zuletzt auf ein Wissen berief, das quer und konträr zur akademischen Wissenschaft lag. Der Protest gegen Risikotechnologien wie die Atomkraft, gegen industriell verursachte Umweltverseuchung oder die atomare Aufrüstung stellte meist zugleich die ‚etablierte‘ Wissenschaft, ihre Institutionen, ihre Autorität und ihren Anspruch als Garanten eines menschlichen und zukunftsfähigen Wissens infrage.

Die Grenzen verliefen dabei nicht trennscharf. Vielfach waren es Wissenschaftler*innen aus dem akademischen Betrieb selbst, die eine solche Sicht mit ihren Informationen und ihrer Haltung untermauerten. Und die Gegenexpert*innen außerhalb der wissenschaftlichen Institutionen erarbeiteten sich mit der Zeit respektable Expertise. Die Wissenschaftsforscherin Helga Nowotny, die die österreichische Staatsdebatte um die Atomkraft im Jahr 1979 beobachtete, attestierte den „kritischen Experten“ profundes Wissen und warnte, dass man den Bemühungen der Gegenbewegungen nicht gerecht werde, wenn man sie als „either anti- or pseudo-science“ abtue (Nowotny 1979a: 110–112; Nowotny 1979b: 9). Der Soziologe Ulrich Beck kam bekanntlich 1986 sogar zu dem Schluss, dass die grassierende „Wissenschafts- und Technikkritik und -skepsis“ angesichts des „Versagens der wissenschaftlich-technischen Rationalität“ und „wachsender Risiken und Zivilisationsgefährdungen“ nur der Anfang einer „reflexiven Modernisierung“ im Verhältnis von Wissenschaft, Öffentlichkeit und Politik sei (Beck 1986: 71 und 78). Die Theoretiker*innen der Wissensgesellschaft und andere Wissensforscher*innen waren offensichtlich damals von der Fundierung und gesellschaftsverändernden Bedeutung des Gegenwissens der Alternativbewegungen überzeugt (vgl. auch Güttler et al. 2016; Stadler et al. 2020b: III/2).

Schon diese Schlaglichter verdeutlichen, dass die Vertrauenskrise, die die Wissenschaft vor gut 50 Jahren erfasste – und nie ganz losgelassen hat –, mit der politischen Vertrauenskrise jener Jahre, wie sie die *Special Section* zu „Political Scientific Activism“ in diesem Heft thematisiert (Germann et al. 2022), verbunden war. Das Unbehagen gegenüber der Technik und die Kritik etwa, mit der die Anti-Atomkraft-Bewegung die Atomwissenschaftler*innen konfrontierte, war mit der Infragestellung der politischen Legitimität des verhassten „Atomstaats“ verbunden. Doch darf man in diesem Zusammenhang nicht vergessen, dass auch jenseits der Bewegungen und ihrer engeren Geschichte Wissensgewissheiten erodierten. So fand die Planungseuphorie, die die Politik in den 1960er Jahren beherrschte, angesichts neuer „Unübersichtlichkeiten“ ein Ende (Seefried 2015; Eberspächer 2019; Faulenbach 2011: 428–449, 569–675). Einhergehend damit endete der Hype der Zukunftswissenschaften, welche sich auf das absehbarere Feld der Technikfolgenabschätzung zurückzogen. Das Gegenwissen befeuerte diese Doppelkrise des Wissens und des Regierens; aber es bot sich auch als Krisenwissen an, das Auswege eröffnete.

Vertrauensverlust und Bruch mit dem herrschenden Wissenschaftsbetrieb kamen nicht nur in der direkten Konfrontation zwischen Gegenexpert*innen und etablierten Wissenschaftler*innen zum Ausdruck, wie sie typisch war für den Protest gegen bestehende Verhältnisse, wie etwa im Fall der Kritik an der Atomkraft und andere Risikotechnologien (Esselborn & Zachmann 2022). Die andere, zukunftsgerichtete Seite des Gegenwissens verkörperte der Aufbruch. Nachdem die revolutionären Träume der 68er-Bewegung mehr oder weniger verflogen waren, sollte wenigstens mit dem Aufbau einer sogenannten Gegengesellschaft, geprägt durch eine „alternative Ökonomie“ oder „Gegenökonomie“, begonnen werden (Reichardt 2014; Falasca et al. 2018). Wo aber anknüpfen, wenn die Instanzen bisheriger Gewissheiten, die Expert*innen und Apologet*innen des wissenschaftlich-technischen Fortschritts, im Mühlrad eines radikalen Wissenschafts- und Technikmisstrauens zerbröselten und man selber den etablierten gesellschaftlichen Institutionen absprach, Antworten auf die drängenden Fragen geben zu können? Das Gegenwissen dieser Jahre war insofern auch Ausdruck und Teil der Hoffnung, dass gesellschaftsverändernde Alternativen möglich waren – auch wenn der Schleier entmutigter Zukunftsgewissheit (vgl. Mende 2017), der nach meinem Eindruck vor allem für spätere Jahre nach 1980 charakteristisch sein sollte, bereits hier oder da die Szenerie bestimmte.

Dass ganz unterschiedliche Wege im Schmelztiegel der Neuen Sozialen Bewegungen denkbar waren und beschritten wurden, wird im Panorama der Menschen, ihrer hochfliegenden Überlegungen und Visionen, waghalsigen Projekte, verschrobenen Wurschteleien oder auch mutigen Unternehmensgründungen schnell deutlich (Stadler et al. 2020b). Dennoch gab es Verbindendes. Die Schlagworte der Stunde jedenfalls lauteten überall ähnlich: Selbermachen! Erfahrungen sammeln! Gemeinsames Lernen! Oder in den Worten von Atomkraftgegner*innen des Jahres 1976: „Wir können also nicht blindlings darauf vertrauen, daß jeder Wissenschaftler sich auf dem richtigen Weg zur Wahrheit befindet. Was wahr und gut für uns ist, müssen wir gemeinsam herausfinden. Dabei trägt jeder Verantwortung“ (Autorenkollektiv 1976: 169). Der Soziologe Harald Glätzer fasste diese Maxime als teilnehmender Beobachter so zusammen:

Die Bereitschaft zum permanenten Lernen und Arbeiten, Einsatz aller Sinne, Offenheit und wissenschaftliches Interesse (nicht eine Seminar-Elfenbeinturm-Wissenschaft) sind Voraussetzungen, um die Erde fruchtbar für alles Leben zu halten (Glätzer 1978: 230).

Auch der Soziologe Walter Hollstein identifizierte in seiner ebenfalls Ende der 1970er Jahre erschienenen und preisgekrönten Zusammenschau „alternativer Lebensformen“ selbst gemachte Erfahrungen als entscheidende Zutat beim Aufbau der utopischen „Gegengesellschaft“. Viele „alternative Projekte, Communities, Landkommunen etc.“ hätten „mittlerweile Erfahrungen“ im Umgang „mit einer menschlicheren und ökologischeren Technik“ gesammelt und damit begonnen, sich systematisch darüber auszutauschen (Hollstein 1979: 125 und 154). Das Wissen zirkuliere in „unzähligen Fibeln, Katalogen, Gebrauchsanweisungen, Therapie- und Erfahrungsberichten“ – meist reichlich illustriert (Hollstein 1979: 140). Die kritischen und alternativen Projekte und Initiativen nahmen dabei ganz unterschiedliche Formen an: als Projekttutorien und kritische Forschungsprojekte innerhalb der Hochschulen, als außerakademische Forschungsinstitute, wie im Fall des Freiburger Öko-Instituts oder des Heidelberger Instituts für Energie- und Umweltforschung (IFEU), als Wissenschaftsläden und Netzwerke, als selbstorganisierte Lehr- und Lernstätten oder in Form der unzähligen einzelnen Initiativen, ihren Akteur*innen und epistemischen Strategien in den Städten und auf dem Land.^2^ Die beachtlich wachsende Infrastruktur kritischer und alternativer Wissensgenerierung und -sammlung, des Erfahrungsaustausches und des Lernens war Ergebnis und sichtbarstes Zeichen der Bedeutung, die die Alternativbewegung den authentischen, nicht aus zweiter Hand der Wissenschaft gelieferten Erfahrungen zuschrieb.

Besinnung auf sich selbst und kollektive Kräfte bedeutete, selbst Teil des Werkzeugkastens der kommenden Gegengesellschaft zu werden. Der Futurologe und Sympathisant der Alternativbewegung Robert Jungk wähnte bereits eine „experimentelle Gesellschaft“ an der Peripherie der Normalgesellschaft im Entstehen, einen neuen Erfahrungsraum fern der etablierten Wissenschaften (Jungk 1973: 131; vgl. ähnlich Schumacher 1974: 215–216) – nicht ganz unähnlich im Übrigen den Konzepten der Wissens- und Informationsgesellschaft, welche zur selben Zeit unabhängig von den Träumen der Alternativen ebenfalls die Wissensschöpfung am Ursprung gesellschaftlicher Erneuerung sahen.

Die wenigen Stimmen aus der zeitgenössischen Empirie und Soziologie des Gegenwissens verdeutlichen schon, wie ernst es die Protagonist*innen der Alternativbewegungen mit der Vorgabe meinten, sich auf ihr eigenes Vermögen und ihre eigenen Stärken als Gemeinschaft zu stützen. Unbeantwortet bzw. umstritten blieb indes die Frage, wie genau die gegenwissentliche Praxis aussehen sollte (vgl. auch Esselborn & Zachmann 2022).

## Fragen und Beiträge dieser Special Section

Die Geschichte der Neuen Sozialen Bewegungen und der Alternativbewegungen in der Bundesrepublik verzeichnet seit Jahren ein wachsendes Interesse. Dabei scheinen die Geschichtswissenschaften bislang übersehen bzw. keine Ausdrucksmöglichkeiten für den Umstand gefunden zu haben, dass die Anliegen der sozialen und alternativen Bewegungen jener Zeit – zum Beispiel Atomkraftwerk-Sicherheit, Vergiftung der Lebensgrundlagen, neue ökologische Lebensformen – meist wissensvermittelte Themen darstellten. Die These, dass die Frage des Wissens keineswegs akzidentiell für die Neuen Sozialen Bewegungen war, sondern zentral und konstitutiv, bildet den Ausgangspunkt dieser *Special Section*. Die Frage lautet, auf welcher Wissensbasis die Protagonist*innen der Alternativbewegungen ihre Kritiken formulierten, ihre Aktivitäten entfalteten und ihre Projekte begründeten. Wie und warum generierten sie kritisches Wissen? Was war das für ein Wissen? Aus welchen Quellen stammte das alternative Wissen, das den Ausweg aus der Krise ebnen sollte? Wie wurde es erzeugt und von wem? Auf wen und was stützten sich die Protagonist*innen des Gegenwissens? Welche Formen nahm das Gegenwissen an und wie zirkulierte es? Nicht zuletzt stellt sich die Frage, ob der Rahmen der Bewegungsgeschichte reicht, um das Gegenwissen zu verstehen?

Die Aspekte, die im Vordergrund der Beiträge dieser *Special Section* stehen, sind Topologie, Zeitlichkeit sowie Kontextualität und Organisation des Gegenwissens:

Der Beitrag Gegenexpert*innen: Umwelt, Aktivismus und die regionalen Epistemologien des Widerstandes von Nils Güttler konzentriert sich auf drei Fallbeispiele aus dem Rhein-Main-Raum. Er fokussiert damit auf die „Geographie der Wissensproduktion“, das heißt die lokale und regionale Situierung ökologischen Gegenwissens im Zusammenhang des Protests gegen die ansässige Chemieindustrie und den Frankfurter Flughafenausbau zwischen den 1960er und 1980er Jahren. Die drei in dem Beitrag vorgestellten Beispiele sind, erstens, die im Kontext des Ausbaus des Flughafens Frankfurt entwickelte interdisziplinär angelegte Lärmforschung, zweitens, die von der Initiative zum Schutz des Rheins und der Grünen-Politikerin Jutta Ditfurth skandalisierte Entsorgung von Industriechemikalien in den Rhein sowie, drittens, die von Studierenden um den Technikphilosophen Gernot Böhme an der TH Darmstadt untersuchte industrieverursachte Senkung des Grundwasserspiegels. Das Zentrum der ökologischen Gegenexpertise lag, wie die Beispiele nahelegen, nicht in den urbanen Zentren, sondern in suburbanen Ballungsräumen.

Gründung und Aktivitäten der Stiftung Ökologischer Landbau (SÖL) stehen im Mittelpunkt des Beitrags *Zeitlichkeit des Gegenwissens in der ökologischen Landbau-Szene (1970–1999) *von Alexander von Schwerin. Der Beitrag untersucht am Beispiel der vom Kaiserslauterner Industriellenehepaar Karl Werner Kieffer und Dagi Kieffer gegründeten SÖL die Bedeutung von Zeitlichkeit bei der Formierung alternativer Wissensbestände. Vor dem Hintergrund, dass die etablierten Landwirtschaftswissenschaften weitgehend der Intensivlandwirtschaft verpflichtet waren, entwickelte sich die SÖL zu einer wichtigen Lobby‑, Multiplikations- und nicht zuletzt Forschungsorganisation für den ökologischen Landbau in der Bundesrepublik. Zentral war dabei der Rückgriff auf die fast vergessene Bodenkunde der 1920er und 1930er Jahre und damit ein dem linearen wissenschaftlich-technischen Fortschrittsdenken entgegengesetztes Modell konservativer Modernisierung.

Der dritte Beitrag *Gründerzeit. Hightech und Alternativen der Wissenschaft in West-Berlin* von Max Stadler befasst sich mit dem aus dem Umfeld der Studierendenschaft der TU Berlin heraus im Jahr 1982 gegründeten Berliner Wissenschaftsladen e. V. (WILAB). Das linksalternative Projekt, welches das Ziel verfolgte, eine Brücke zwischen Bewegung und Universität zu schlagen, stellt eine nicht zufällige Parallele zur West-Berliner Hochschul- und Wirtschaftspolitik dar, die zur gleichen Zeit den Elfenbeinturm unter dem Schlagwort Technologietransfer für die Dienste an der Gesellschaft stärker durchlässig zu machen trachtete. Im Zentrum stehen der Einzug des Computers in die Arbeitswelt und die Informationswissenschaften, um deren Nutzwert für die gesellschaftliche Entwicklung in diesen Jahren eine Art Wettlauf entstand. Die unternehmerisch durchaus ambitionierten Alternativprojekte stellen sich in diesem Zusammenhang weniger als die unbeabsichtigten Helfershelfer bei der technologischen Erneuerung der Berliner Wirtschaft dar als die Ausläufer der vorausgegangenen Reformära, die mit ihren organisatorischen Möglichkeiten in den 1980er Jahren an ihre Grenzen stießen.

## Ergebnisse und Ausblick: Fragen der Definition und Eingrenzung

Die in dieser *Special Section* versammelten Beiträge zeigen schlaglichtartig,

1. dass alternative Wissensinhalte, ihre Herstellung und Organisation offenbar eine konstitutive Rolle für die Formation der Neuen Sozialen und alternativen Bewegungen und ihr politisches Handeln spielten,
2. dass die Protagonist*innen dieser Bewegungen eigene Strukturen und Formen der Organisation, Erzeugung, des Managements und der Verbreitung alternativen Wissens entwickelten,
3. dass sich die alternativen Wissensinhalte in der Art sehr unterschieden und in bislang kaum erforschter Weise zustande kamen,
4. dass die Geschichte des Gegenwissens über die Geschichte der Bewegung hinausgeht,
5. dass keine scharfe Trennung zwischen der „etablierten“ Wissenschaft und den Entstehungs- und Wirkungsbereichen des Gegenwissens bestand,
6. dass das „Gegenwissen“ aber die etablierten wissenschaftlichen Institutionen herausforderte, teils aus ihrem Inneren heraus, teils von sehr exzentrischen Positionen aus.

Die Spannung zwischen alternativem und akademischem Wissen scheint überhaupt ein prägendes Charakteristikum des hier empirisch umrissenen Phänomens zu sein. Das Beispiel einer industrialisierten Region wie dem Rhein-Main-Gebiet erhellt nicht nur die Geschichte regionaler Umweltinitiativen. Deutlich werden die Interdependenzen zwischen Ökoaktivismus und Umweltwissenschaften. Im Problembewusstsein der Gegenexpert*innen brach sich das Regionale im Austausch mit den Umweltwissenschaften Bahn. Die Interdependenzen verdichteten sich förmlich im „Darmstädter Paradigma“ als programmatischer Zusammenhang von Wissenschaft, Technik und Natur in einer zukünftigen postindustriellen Gesellschaft. Im Beispiel des ökologischen Landbaus wiederum konstituierte sich das Gegenwissen als ein Zukunftswissen, das aus der Rückbesinnung auf verschüttete oder unentdeckte Wissensbestände schöpfte, im Gegensatz zu einem auf die Gegenwart der Atomgefahren bezogenen Risikowissen. Während manche fernab der westlichen Zivilisation fündig wurden, brauchten sich die ökologischen Landbau-Pionier*innen nur in der Zeit zurückzubewegen. Deutlich wird zugleich der Einfluss einer etablierten Elite aus Wirtschaft und Wissenschaft auf die Etablierung des ökologischen Landbaus, welcher die Offenheit gegengesellschaftlicher Utopie wiederum einschränkte. Im Fall des West-Berliner Wissenschaftsladens standen die Akteure des Gegenwissens unter Eindruck der Modernisierung der regionalen Industrie, welche Politik und Wirtschaft des Bundeslandes mit Unterstützung der Wissenschaft mit Macht vorantrieben. Ihr Projekt verfolgte eine kritische Ausrichtung der Informationswissenschaften an der TU Berlin und trat damit in Widerspruch zu den Vorstellungen der TU-Leitung, wie Wissenschaft dem gesellschaftlichen Nutzen dienen sollte. Dabei drohten die unternehmerischen Formen des Gegenwissens, sich den offiziellen Modernisierungsplänen anzugleichen oder schlimmstenfalls Vorbilder für diese zu liefern, wie die zeitgenössischen Akteur*innen selbst problematisierten.

Eine systematische Geschichte, die vergleichend syn- und diachron andere Zeiten und Schauplätze des Gegenwissens und die mit ihr verbundenen Spannungsverhältnisse zwischen den etablierten Institutionen der Wissenserzeugung und ihren inneren und äußeren Kritiker*innen bzw. Alternativen in den Blick nimmt, steht aus und würde voraussetzen, Definitions- und Abgrenzungsfragen zu klären, am besten bereits auf Grundlage solcher Fallbeispiele.

Vorläufig kann „Gegenwissen“ als gesellschaftliche Absetzbewegung verstanden werden, in der die Kritik an einer als vorherrschend verstandenen Wissenschaft, Medizin oder Technik und die Suche nach Alternativen mit der Ausprägung eigener Wissensinhalte und -formen, Strukturen oder epistemischer Praxen einherging. Ein entscheidendes Merkmal – nicht zuletzt Abgrenzungsmerkmal gegenüber exzentrischeren Wissensformen – liegt in seiner Relationalität. Das Gegenwissen konstituierte sich *im Kern* nicht in Abkehr und im Abbruch, sondern in einem, durch Austausch und Auseinandersetzung geprägten Spannungsverhältnis zu Wissenschaft, Technik und Medizin. Dementsprechend beschrieb Nowotny die Beziehung zwischen den von ihr beobachteten „Counter-movements“ und den Wissenschaften scharfsinnig als „field of conflict“ und „antagonistic interactions“ (Nowotny 1979b: 2–4).

Fließende Übergänge prägten zugleich das Verhältnis nicht nur zur herrschenden Wissenschaft, Medizin und Technik als dem einen Pol, sondern auch zu esoterischen, durch Entkopplung vom wissenschaftlichen Diskurs gekennzeichneten Positionen am anderen Pol. Einerseits: Lange, bevor die Umweltbewegung die Chemieindustrie als ihren Feind ausmachte, lieferten „kritische“ Wissenschaftler*innen, wie Heiko Stoffs Geschichte des Verbraucherschutzes etwa zeigt, das Wissen über die Gefahren der Chemisierung der menschlichen Umwelt (Stoff 2015). Wissenschaftler*innen spielten, so Joachim Radkau, dann auch eine führende Rolle in der Entstehung der Umweltbewegung: „eine neue Elite, selbstbewusst und wohlinformiert“, deren Stellung im wissenschaftlichen Establishment allerdings nicht eindeutig geklärt war (Radkau 2011: 151 und 154). In letzter Konsequenz war das Gegenwissen nicht an einen spezifischen gesellschaftlichen Ort gebunden, auch wenn die sozialen Bewegungen ihm sein besonderes historisches Gepräge in der in dieser *Special Section* im Vordergrund stehenden Epoche verliehen. Schon früh ist auf alternative Ursprünge kritischen Umweltbewusstseins und ökologischer Initiative jenseits des Rahmens sozialer Bewegungen hingewiesen worden, gesellschaftliche Eliten und (supra)staatliche Akteure bis hin zur NATO eingeschlossen.^3^ Damit stellen sich natürlich allgemeine Fragen zur Abgrenzung. Andererseits: Am anderen Pol des Spannungsverhältnisses lauerte der Übergang zum Irrationalismus. Die Kritik an den Wissenschaften und der „westlichen Vernunft“ mündete dann im Abbruch von Austausch und Auseinandersetzung mit den Wissenschaften und in der Orientierung an radikal anderen Wissenszusammenhängen, indigenen Kulturen, spirituellen Traditionen oder den Hexen, Schamanen und Kelten der Vergangenheit, durchaus auch inspiriert von „abtrünnigen“ Naturwissenschaftler*innen, wie die Beispiele von Fritjof Capra oder Francisco Varela vorführen (Stadler et al. 2020b: II/2–25).

Nur wenn die historische Forschung jenen fließenden Übergängen beidseitig genau folgt, wird sie vermutlich einen Beitrag zu den Formationsbedingungen *gegen*wissenschaftlicher und *anti*wissenschaftlicher Wissenszusammenhänge heutiger Tage und deren Unterscheidung leisten können. Und wie das Beispiel von Helga Nowotny zeigt, führt das Thema des Gegenwissens die Wissenschaftsgeschichte auch in ihre eigene Vergangenheit zurück, in der die Bedingungen und die Gemachtheit von Wissen zum intensiven Thema der Forschung und/oder politischen Arbeit wurden.

## Footnotes

1 Ein weiterer Beitrag auf dieser Tagung von Anna Maria Schmidt, Universität Duisburg-Essen, ist bereits an anderer Stelle erschienen: Schmidt (2019).

2 Zu einer Auflistung von Projekten zum Erfahrungsaustausch siehe Beer (1983: 27).

3 Küppers et al. (1979); siehe auch Radkau (2011: 140 und 143); zur NATO siehe Turchetti (2019).
